# Antimicrobial activities of fungus comb extracts isolated from Indomalayan termite (*Macrotermes gilvus* Hagen) mound

**DOI:** 10.1186/s13568-022-01359-0

**Published:** 2022-02-10

**Authors:** Lucia Dhiantika Witasari, Khairunnasa Wizdjanul Wahyu, Bonifasia Junita Anugrahani, Dina Clarissa Kurniawan, Aris Haryanto, Dodi Nandika, Lina Karlinasari, Arinana Arinana, Irmanida Batubara, Djoko Santoso, Yanti Rachmayanti, Dikhi Firmansyah, I Ketut Sudiana, Decsa Medika Hertanto

**Affiliations:** 1 Department of Food and Agricultural Product Technology, Faculty of Agricultural Technology, Gadjah Mada University, Bulaksumur, Yogyakarta, 55281 Indonesia; 2 Faculty of Veterinary Medicine, Gadjah Mada University, Bulaksumur, Yogyakarta, 55281 Indonesia; 3 Department of Forest Products, Faculty of Forestry and Environment, IPB University, Darmaga Campus, Bogor, 16680 West Java Indonesia; 4 Department of Chemistry, Faculty of Mathematics and Natural Sciences, Tropical Biopharmaca Research Center, IPB University, Darmaga Campus, Bogor, 16680 West Java Indonesia; 5 Faculty of Medicine, Campus A Universitas Airlangga, Surabaya, 60132 East Java Indonesia; 6 Department of Chemistry, Faculty of Mathematics and Natural Sciences, Institut Teknologi Bandung, Bandung, 40132 West Java Indonesia

**Keywords:** *Macrotermes gilvus* Hagen, Fungus comb extracts, Antibacterial, Antifungal

## Abstract

Incorporating antimicrobial components into food packaging materials can prevent microbial contamination. Fungus combs could be an alternative source of natural antimicrobial agents. In this study, *n-*hexane, ethyl acetate, methanol, and water extracts were obtained from fungus combs isolated from Indomalayan termite (*Macrotermes gilvus* Hagen) mound. Their antibacterial and antifungal activities against food spoilage microorganisms including *Escherichia coli* ATCC 25922, *Pseudomonas aeruginosa* *AT*CC 27853, *Staphylococcus aureus ATCC *25923, *Aspergillus flavus*, and *Aspergillus niger* were evaluated by Kirby–Bauer disc diffusion and microdilution. Results showed that ethyl acetate extract formed the largest diameter inhibition zone for all tested bacteria and fungi, exhibited antibacterial activity against all tested bacteria with minimum inhibitory concentration (MIC) and minimum bactericidal concentration (MBC) values of 0.39 and 0.78 mg/mL, respectively, and suppressed *A. flavus* and *A. niger* with an MIC value of 0.78 mg/mL. This extract contained guaiacol and syringol, which were predicted as the main antimicrobial components in fungus comb. *n-*Hexane extract only inhibited Gram-positive bacteria. *S. aureus ATCC *25923 was the most sensitive to all the extracts, and *A. flavus* was more sensitive than *A. niger*. All these fungus comb extracts exhibited antimicrobial activity against *E. coli* ATCC 25922, *P. aeruginosa* ATCC 27853, *S. aureus* ATCC 25923, *A. flavus,* and *A. niger*. This study revealed that fungus comb extracts, especially ethyl acetate, could be considered as a new antimicrobial agent.

**Graphical Abstract:**

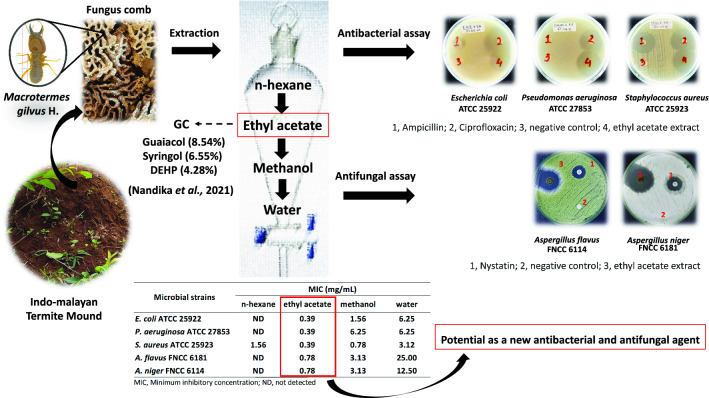

**Supplementary Information:**

The online version contains supplementary material available at 10.1186/s13568-022-01359-0.

## Keypoints

- Ethyl acetate extract from fungus combs exhibited high antimicrobial activity against *Staphylococcus aureus* ATCC 25923, *Escherichia coli* ATCC 25922, *Pseudomonas aeruginosa* ATCC 2785, *Aspergillus flavus* FNCC 6181, and *Aspergillus niger* FNCC 6114.
- Ethyl acetate extract from fungus combs contained phenolic compounds such asguaiacol and syringol, which are predicted as the main antimicrobial substances.
- *Staphylococcus aureus* ATCC 25923 was the most sensitive against *n*-hexane, ethyl acetate, methanol, and water extracts from fungus comb

**Supplementary Information:**

The online version contains supplementary material available at 10.1186/s13568-022-01359-0.

## Introduction

Fruits and vegetables are essential components of a healthy diet; however, produce has recently been associated with diverse foodborne illness outbreaks in many countries. Fruits and vegetables are usually acidic and hence resistant to invasion by bacteria and fungi, especially *Aspergillus flavus* and *Aspergillus niger* for corn, tomatoes, grape, strawberries, figs, peaches, pears, apples, citrus, melons, and mangoes (Bui-Klimke and Wu 2015). *A. flavus* is a well-recognized producer of aflatoxin, and *A. niger* is known as ochratoxin producer (Pfliegler et al. 2020). In addition, fresh herbs such as basil, parsley, and leafy greens, especially spinach and lettuce, are potential sources of bacterial contamination (FAO/WHO 2008). Denis et al. (2016) reported bacterial infections from *Escherichia coli*, *Salmonella*, and *L.* monocytogenes in retailed fresh vegetables and fruits in Canada. Other pathogenic bacteria that often contaminate food are *Staphylococcus aureus* and *Pseudomonas aeruginosa*.

The microbial attack on food products can occur during pre-harvest, transportation, storage, and food processing (Bbosa et al. 2013). In tropical countries, contamination risk during postharvest is higher than in the field due to warm and humid environments. Therefore, maintaining low temperature at 2–3 °C and relative humidity around 90% and 95% is essential to avoid decay-causing fungi (Boer et al. 2009). Although effort has been devoted to adjust the environment, some fruits and vegetables have a high susceptibility to mechanical damage during transportation and a great chance of atmospheric damages due to decreasing oxygen and increasing carbon dioxide levels. According to Feliziani and Romanazzi (2016), consumer request has increased significantly for new technologies of safe and environmentally friendly fruit and vegetable packaging, such as edible or biodegradable coatings. In addition to acting as a barrier to mechanical injury, the coating can be injected by antimicrobial agents that inhibit the growth of food spoilage fungi and bacteria. Therefore, the use of antimicrobial agents in food packaging has become a potential solution for preventing postharvest loss.

Natural products are the most abundant source of antimicrobials. Among which, fungus combs can minimize potentially pathogenic fungi and are competitive in the environment to maintain the health of their colonies (Arango 2015). These special structures are created by termite colonies from the sub-family Macrotermitinae (Isoptera: Termitidae) in their nests as a substrate for the growth of only one fungus species, *Termitomyces* sp. (Arshad and Schnitzer 1987; Arinana et al. 2016). Fungus combs have chemical compounds might suppress the growth of another species and therefore are a potential source of active compounds for an organic antimicrobial agent. Nandika et al. (2021) extracted the chemical components of a fungus comb from Indomalayan termite (*Macrotermes gilvus* Hagen) (Isoptera: Termitidae) and identified them as phenol, hydroquinone, steroids, terpenoids, and saponin compounds. In addition, the ethyl acetate extract inhibited the growth of *Aspergillus foetidu*s, a fungus that attacks wooden raw materials, including rubberwood (*Hevea brasiliensis* Muell. Arg.). However, the bioactivity of fungus comb extract from Indomalayan termite (*M. gilvus* Hagen) mounds as an antifungal and antibacterial agent has not been reported. In the present research, the antifungal and antibacterial activities of fungus comb extracts against *A. flavus*, *A. niger*, *E. coli* ATCC 25922, *P. aeruginosa* ATCC 27853, and *S. aureus* ATCC 25923 were examined.

## Material and methods

### Fungus comb preparation

Fungus comb extracts were obtained from Indomalayan termite (*M. gilvus* Hagen) (Isoptera: Termitidae) mounds in Yanlappa Experimental Forest, Bogor, West Java Province by using four different solvents (*n-*hexane, ethyl acetate, methanol, and water) (Nandika et al. 2021). The extraction yields of *n-*hexane, ethyl acetate, methanol, and water extracts were 0.09%, 1.73%, 2.53%, and 4.61%, respectively. A sample of 0.5 mg of solid extract was dissolved in 500 mL of its solvent.

### Bacterial culture

*E. coli* ATCC 25922, *P. aeruginosa AT*CC 27853, and *S. aureus ATCC* 25923 cultures were grown on nutrient agar (OXOID, Basingstoke, England) media, and then incubated at 37 °C for 18 h. Culture suspensions of *E. coli* ATCC 25922, *S. aureus* ATCC 25923, and *P. aeruginosa* ATCC 27853 were prepared using a physiological solution containing 0.85% NaCl. The bacterial suspensions were then compared with standard McFarland 0.5 solution (1.5 × 10^8^ CFU/mL).

### Antibacterial susceptibility assay

Antibacterial susceptibility was detected by disc diffusion in accordance with the standards set by the Clinical Laboratory Standard Institute (CLSI). In brief, 100 µL of an overnight culture was diluted in saline solution to ~ 1.5 × 10^8^ CFU/mL (0.5 McFarland turbidity standard). This suspension was flooded into Mueller–Hinton agar (Oxoid Ltd., Basingstoke, UK). The sample’s paper disc was dispensed into the inoculated plate and then incubated at 37 °C for 24 h. The diameters of the clear zones around each paper disc were measured after incubation. Each extract was tested in triplicate.

### Determination of minimum inhibitory concentration (MIC) and minimum bactericidal concentration (MBC)

MIC and MBC were determined by microdilution in accordance with CLSI. Each well on the 96-well microplate (BIOLOGIX, Europe) was filled with Mueller–Hinton broth (HiMEDIA, India) media (100 µL), suspension of *E.coli* ATCC 25922, *P. aeruginosa* ATCC 2785, *S. aureus* ATCC 25923 (each 10 µL, 1.5 × 10^8^ CFU/mL), and fungus comb extract (100 µL). Each well has a different concentration of fungus comb extract, starting from a concentration of 100–0.1 mg/mL. A medium added with bacterial suspension was used as a positive control, and pristine broth medium was used as a negative control. The microplate was incubated at 37 °C for 18 h, and the MIC values were measured on the basis of turbidity. A turbid solution indicates the presence of bacterial growth. Afterward, the solutions in the microplate were used as samples to determine the MBC value. The samples with different turbidities from clear to turbid were streaked on Mueller–Hinton agar (OXOID, United Kingdom) media and then incubated for 18 h at 37 °C. The MBC value was determined as the lowest concentration showing no visible bacterial growth.

### Fungal culture

Strains of *A. flavus* FNCC 6181 and *A. niger* FNCC 6114 were grown on potato dextrose agar (MERCK, United States) media, incubated at 30 °C for 48 h, and stored in the refrigerator. Prior to the assay, the concentration of the culture was adjusted to 10^6^ CFU/mL counted using a hemocytometer. The culture was resuspended in 1 mL of 0.05% Tween 80.

### Antifungal susceptibility assay

The antifungal activity of fungus comb extracts against *A. flavus* FNCC 6181 and *A. niger* FNCC 6114 cultures was examined by Kirby–Bauer disc diffusion using 6 mm diameter filter paper discs (OXOID, United Kingdom). First, the cultures were diluted in 1000 µL of 0.05% Tween solution to prepare a homogenous single-celled suspension, which was then inoculated by streak/spread method on potato dextrose agar (MERCK, United States) medium using sterile cotton buds. The test was carried out with three extract concentrations: 2.5, 25, and 50 mg/mL. Antifungal susceptibility was determined by measuring the zone of inhibition (mm) after 48 h of incubation at 30 °C. The samples were compared with an antifungal agent, 100 units of nystatin (OXOID, United Kingdom), as a positive control. Each extract was tested in triplicate.

### Determination of MIC and minimum fungicidal concentration (MFC)

MIC was recorded as the lowest concentration of drug permitting the growth of no spores after 48 h of incubation at 30 °C for each fungus comb extract determined by its turbidity. Its determination was carried out by microdilution using a Roswell Park Memorial Institute broth medium (GIBCO, United States) with l-glutamine, without sodium bicarbonate (NaHCO_3_) supplemented with 2% glucose, buffered to pH 7.0 with 4-(2-hydroxyethyl)-1-piperazineethanesulfonic acid (HEPES) in a 96-well microplate (BIOLOGIX, Europe). A turbid well indicates the presence of fungal growth and vice versa. Each well was filled with 100 µL of broth medium and 100 µL of samples, which were then serially twofold diluted to ensure that every well has a different concentration from 100 mg/mL to 0.1 mg/mL. Afterward, 10 µL of suspension of *A. flavus* FNCC 6181 (10^6^ CFU/mL) and *A. niger* FNCC 6114 (10^6^ CFU/mL). Turbidity was compared with that of the positive control wells containing broth media and fungus culture suspension (10^6^ CFU/mL) and negative control wells containing broth media. The experiment was performed four times. MFC was determined by subculturing the samples from previous MIC value determination. Each well with different turbidities from clear to turbid were streaked on potato dextrose agar medium (MERCK, United States) and then incubated at 30 °C for 48 h. The assay was performed in triplicate. MFC was recorded as the lowest fungus comb extract concentration showing no visible growth of *A. flavus* FNCC 6181 and *A. niger* FNCC 6114.

## Results

### Antibacterial activity

**Fig. 1:**
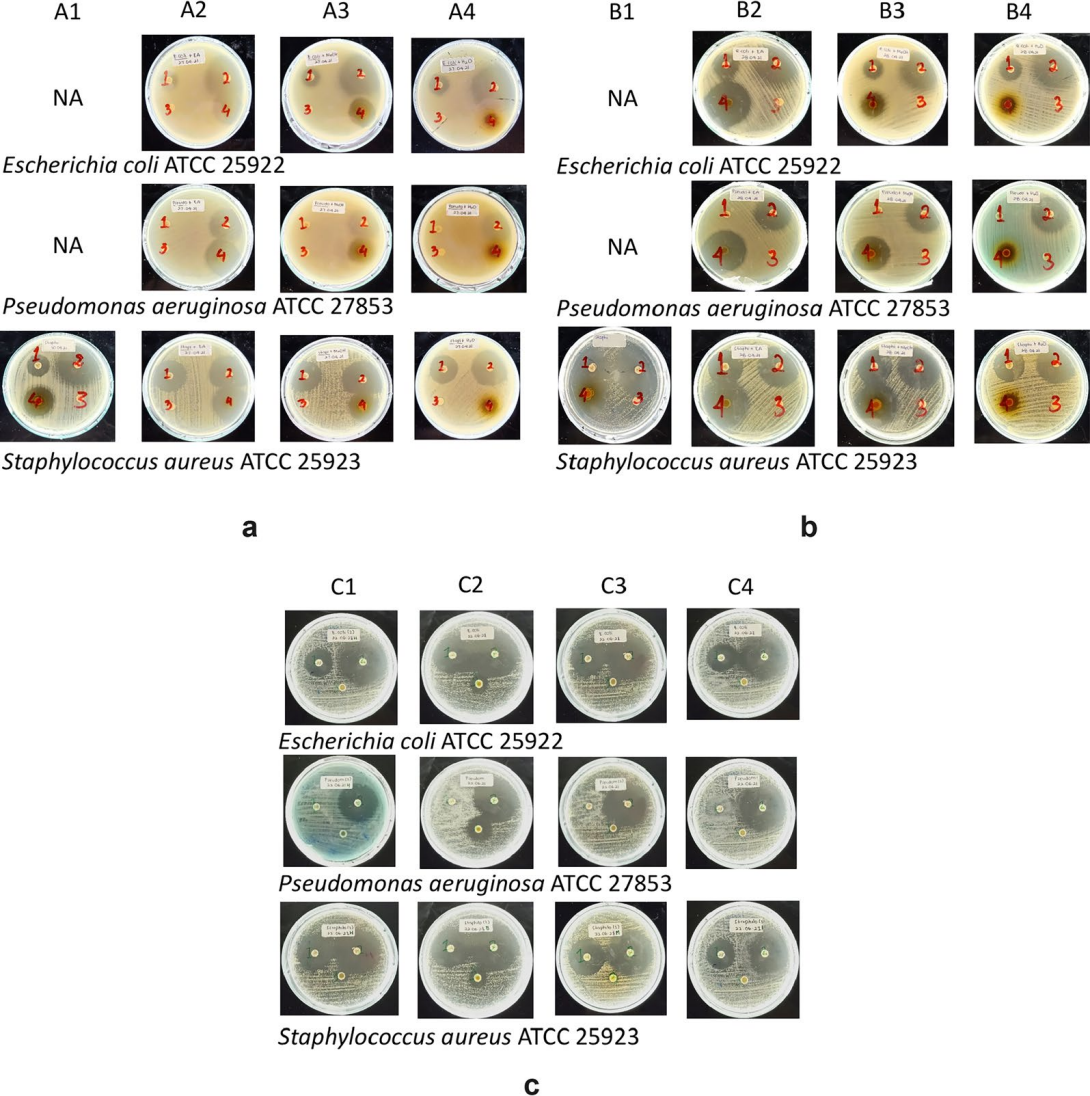
Inhibitory activity of fungus comb extract against *E. coli* ATCC 25922, *P. aeruginosa AT*CC 27853 and *S. aureus ATCC *25923*.* A1, B1, C1, *n-*hexane extract; A2, B2, C2, ethyl acetate extract; A3, B3, C3, methanol extract; A4, B4,C4, water extract. **a** 50 mg; **b** 25 mg; **c** 2.5 mg; 1, Ampicillin 10 μg; 2, Ciprofloxacin 5 μg; 3, negative control (solvent); 4, fungus comb extracts

The antibacterial susceptibility of fungus comb extracts against Gram-negative (*E. coli* ATCC 25922 and *P. aeruginosa* ATCC 2785) and Gram-positive (*S. aureus* ATCC 25923) bacteria was examined by Kirby–Bauer agar diffusion. The diameter inhibition zones (DIZs) resulting from exposure to *n-*hexane, ethyl acetate, methanol, and water extracts of fungus comb are shown in Table 1. The clear zones on bacteria tested with fungus comb extracts are presented in Fig. 1.

**Table 1:** Antibacterial susceptibility assay

Bacteria strains	Diameter of inhibition zone (DIZ) (mm)													
	Fungus comb extract												Positive control	
	*n*-Hexane			Ethyl acetate			Methanol			Water			Amp 10 μg	Cipro 5 μg
	2.5 mg	25 mg	50 mg	2.5 mg	25 mg	50 mg	2.5 mg	25 mg	50 mg	2.5 mg	25 mg	50 mg		
Gram-negative														
*E. coli *ATCC 25922	ND	NA	NA	9.85 ± 0.07	28.20 ± 1.56	36.75 ± 1.20	7.25 ± 0.07	19.20 ± 2.83	21.9 ± 1.41	ND	10.55 ± 0.78	10.65 ± 0.64	25.35 ±0.07	36.05 ±0.07
*P. aeruginosa *ATCC 25923	ND	NA	NA	8.20 ± 0.14	27.65 ± 0.64	32.65 ± 3.46	8.25 ± 0.07	15.05 ± 1.48	31.25 ± 1.34	ND	11.60 ± 0.85	13.10 ± 3.11	ND	29.05 ± 0.21
Gram-positive														
*S. aureus *ATCC 25923	12.95 ± 0.21	18.45 ± 0.35	19.40 ± 0.14	13.75 ± 0.64	24.95 ± 1.63	36.75 ± 1.63	8.55 ± 0.49	21.45 ± 0.21	33.10 ± 1.41	ND	9.25 ± 1.34	13.25 ± 1.20	26.75 ± 0.07	32.15 ± 0.07

Diameter of inhibition zone (mm) presented as means (± SD), comprising a 6 mm paper disk; positive controls were Ampicillin (Amp,10 μg), Ciprofloxacin (Cipro, 5 μg); ND, not detected; NA, not available; three times replication

A high dosage of extracts resulted in a large DIZ in all tested bacteria. Ethyl acetate extract formed the largest DIZ against all tested bacteria. *S. aureus* ATCC 25923 was the most sensitive to all the extracts. Owing to the limitation of *n-*hexane extract, the DIZ data of high doses are unavailable for *E. coli* ATCC 25922 and *P. aeruginosa* ATCC 2785. Nevertheless, this extract in the lowest dose inhibited *S. aureus ATCC *25923 but no other bacteria (Fig. 1c). Ampicillin and ciprofloxacin were used as the positive control. Ciprofloxacin in a lower dosage than the fungus comb extracts showed strong inhibition against all tested bacteria. Ampicillin inhibited only *E. coli* ATCC 25922 and *S. aureus* (Table 1).

Ethyl acetate extract showed the lowest MIC of 0.39 mg/mL and MBC of 0.78 mg/mL in all tested bacteria. *n-*Hexane extract had MIC of 1.56 mg/mL and MBC of 3.13 mg/mL only for Gram-positive bacteria (Table 2, Additional file 1: Figure S1, Figure S2).

**Table 2:** MIC and MFC value of fungus comb extract against bacterial strains

Bacteria strains	Fungus comb extract							
	*n*-Hexane		Ethyl acetate		Methanol		Water	
	MIC (mg/mL)	MBC (mg/mL)	MIC (mg/mL)	MBC (mg/mL)	MIC (mg/mL)	MBC (mg/mL)	MIC (mg/mL)	MBC (mg/mL)
Gram negative bacteria								
*E.coli* ATCC 25922	ND	ND	0.39 ± 0	0.78 ± 0	1.56 ± 0	3.13 ± 0	6.25 ± 0	12.5 ± 0
*P. aeruginosa* ATCC 27853	ND	ND	0.39 ± 0	0.78 ± 0	6.25 ± 0	12.5 ± 0	6.25 ± 0	12.5 ± 0
Gram positive bacteria								
*S. aureus* ATCC 25923	1.56 ± 0	3.13 ± 0	0.39 ± 0	0.78 ± 0	0.78 ± 0	1.56 ± 0	3.13 ± 0	6.25 ± 0

*ND* not detected, three times replication

### Antifungal activity

**Fig. 2:**
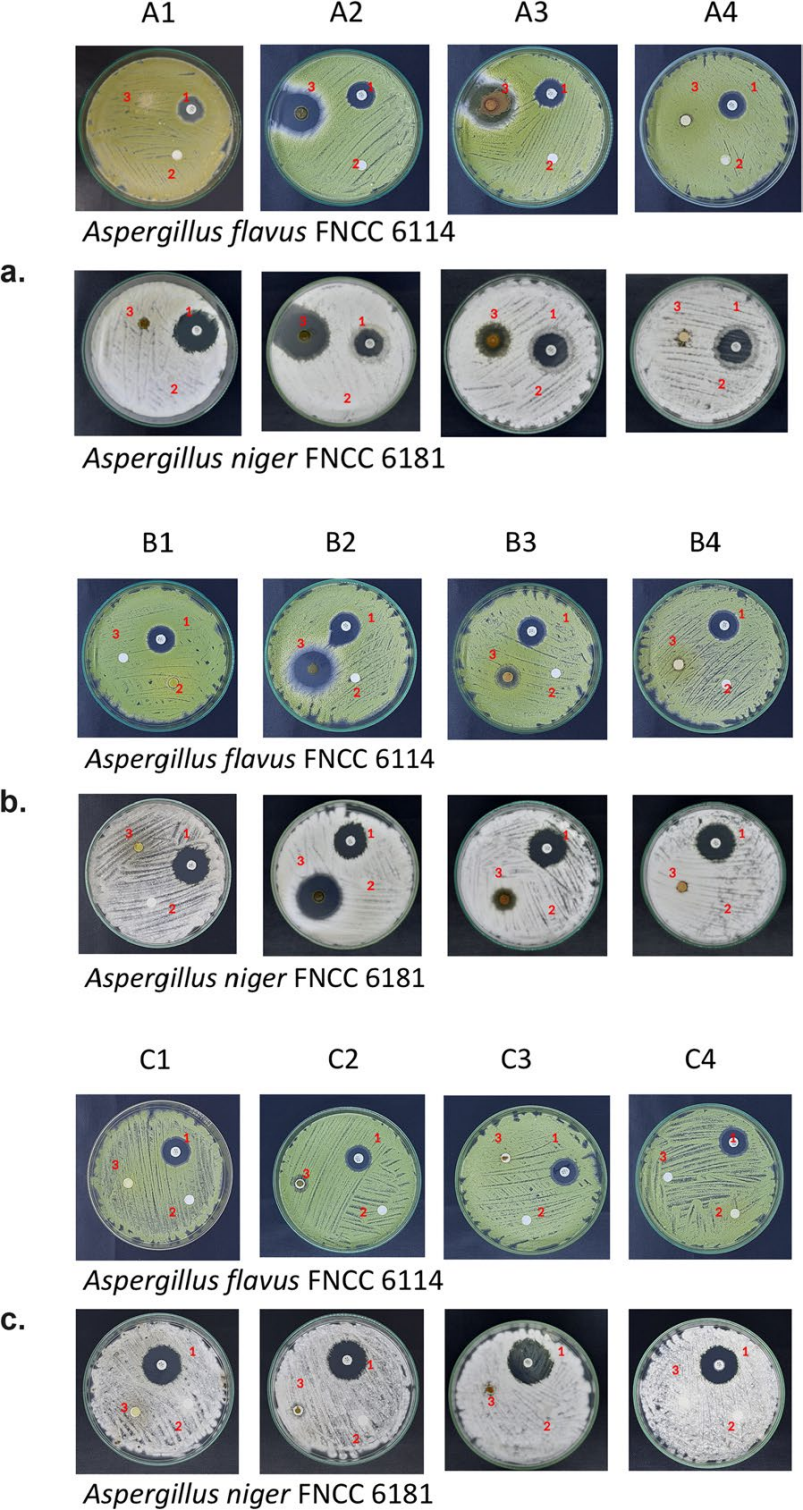
Inhibitory activity of fungus comb extract against *A. flavus* FNCC 6181 and *A. niger* FNCC 6114*.* A1, B1, C1, *n-*hexane extract; A2, B2, C2, ethyl acetate extract; A3, B3, C3, methanol extract; A4, B4,C4, water extract. a, 50 mg; b, 25 mg; c, 2.5 mg; 1, positive control (Nystatin); 2, negative control (solvent); 3, sample fungus comb extract

The antifungal activity of fungus comb extracts against *A. flavus* FNCC 6181 and *A. niger* FNCC 6114 as determined by the clear zone diameter (mm) was examined by Kirby–Bauer disc diffusion (Table 3 and Fig. 2). Ethyl acetate extract generated the largest DIZ against *A. flavus* FNCC 6181 and *A. niger* FNCC 6114 (Table 3). Methanol and water extracts also inhibited both fungi at high dosages (25 and 50 mg). Unfortunately, both fungi were resistant to *n-*hexane extract. Nystatin at 100 U dosage was used as the positive control.

**Table 3:** Antifungal susceptibility assay

Fungi strains	Diameter of inhibition zone (DIZ) (mm)												
	Fungus comb extract												Positive control
	*n*-Hexane			Ethyl acetate			Methanol			Water			Nystatin 100 U
	2.5 mg	25 mg	50 mg	2.5 mg	25 mg	50 mg	2.5 mg	25 mg	50 mg	2.5 mg	25 mg	50 mg	
*A. flavus* FNCC 6181	ND	ND	ND	6.67 ± 0.58	45.67 ± 11.06	49.33 ± 13.43	ND	21.33 ± 7.51	23.67 ± 5.51	ND	9 ± 0	11.67 ± 3.21	17.44 ± 2.13
*A. niger* FNCC 6114	ND	ND	ND	9 ± 10	24 ± 2.65	37.33 ± 6.43	ND	19.67 ± 2.52	22.33 ± 4.73	ND	7.33 ± 1.53	8.67 ± 0.58	24.31 ± 4.24

Diameter of inhibition zone (mm) presented as means (± SD), comprising a 6 mm paper disk; positive controls were Nystatin 100 unit; ND, not detected; three times replication

The results of MIC and MFC investigation supported those from the antifungal susceptibility assay. The lowest MIC and MFC values against *A. flavus* FNCC 6181 and *A. niger* FNCC 6114 were observed for ethyl acetate extract. The MIC and MFC values for both fungi were not detected for *n-*hexane extract (Table 4, Additional file 1: Figure S3, Figure S4).

**Table 4:** MIC and MFC value of fungus comb extract against fungi strains

Fungi strains	Fungus comb extract							
	n-Hexane		Ethyl acetate		Methanol		Water	
	MIC (mg/mL)	MFC (mg/mL)	MIC (mg/mL)	MFC (mg/mL)	MIC (mg/mL)	MFC (mg/mL)	MIC (mg/mL)	MFC (mg/mL)
*A. flavus* FNCC 6181	ND	ND	0.78 ± 0	1.56 ± 0	3.13 ± 0	6.25 ± 0	25 ± 0	25 ± 0
*A. niger* FNCC 6114	ND	ND	0.78 ± 0	1.56 ± 0	3.13 ± 0	3.13 ± 0	12.50 ± 0	12.50 ± 0

ND, not detected, three times replication

## Discussion

This study reported the bioactivity of fungus comb extracts from Indomalayan termite (*M. gilvus* H.) mounds as an antifungal and antibacterial agent against food spoilage microorganisms. The antibacterial activity of fungus comb extracts was tested against *E. coli* ATCC 25922, *P. aeruginosa* ATCC 27853, and *S. aureus* ATCC 25923, and their antifungal activity was examined against *A. flavus* FNCC 6181 and *A. niger* FNCC 6114.

The bacterial inhibitory action of fungus comb extracts differs considerably among species. Ethyl acetate extract generated the largest DIZ against the tested Gram-positive and -negative bacteria, followed by methanol and water extracts (Table 1 and Fig. 1). Water extract inhibited all tested bacteria at a high dosage but not at its lowest dosage. *S. aureus* ATCC 25923 was the most sensitive to all the extracts. *n-*Hexane extract inhibited only Gram-positive bacteria, which are generally more sensitive than Gram-negative bacteria due to their lack of outer membrane (lipopolysaccharide and lipoprotein). In addition, their cell wall architecture tolerates hydrophobic molecules and allows them to easily permeate into the cells (Nazzaro et al. 2013).

Microdilution was used to determine MIC (the smallest concentration of antimicrobials to inhibit microorganism growth) and MBC (the smallest concentration that can inhibit microorganism growth and kill microbes characterized by the absence of colonies on agar media after scraping from each microplate well after incubation) (Migliato et al. 2010). The results showed that ethyl acetate extract exhibited significant antibacterial activity against *E. coli* ATCC 25922, *P. aeruginosa* ATCC 2785), and *S. aureus* ATCC 25923 with MIC and MBC values of 0.39 and 0.78 mg/mL, respectively (Table 2, Additional file 1: Figures S1, S2).

Comparable with antifungal agents such as nystatin, fungus comb extracts showed positive inhibition (Table 3 and Fig. 2). The highest antifungal activity was observed from ethyl acetate extract, followed by methanol. Water extract showed the lowest antifungal activity, and *n-*hexane extract did not inhibit *A. flavus* FNCC 6181 nor *A. niger* FNCC 6114. The largest DIZ was generated by ethyl acetate at 50 mg of dosage against *A. flavus* FNCC 6181. These results showed that *A. flavus* FNCC 6181 was more sensitive than *A. niger* FNCC 6114 (Table 3). The lowest MIC and MFC values were observed for ethyl acetate extract against *A. flavus* FNCC 6181 and *A. niger* FNCC 6114 (Table 4).

The chemical composition of ethyl acetate extract from fungus combs was previously analyzed by gas chromatography (Nandika 2021) (Additional file 1: Figure S5, Table S1). The dominant compounds were glycerol (28.93%), phenol, 2-methoxy- (8.54%), phenol, 2,6-dimethoxy- (6.55%), and bis(2-ethylhexyl) phthalate (DEHP) (4.28%). Meanwhile, the as major compounds of *n-*hexane extract consisted of DEHP (69.43%), methyl palmitate (4.55%), methyl oleate (4.17%), methyl linolelaidate (2.03%) and benzenepropanoic acid, 3,5-bis(1,1 dimethylethyl)-4-hydroxy-,methyl ester (1.16%) (Additional file 1: Figure S5, Table S2).

Among these compounds, DEHP is an ester of phthalic acid and naturally synthesized by plants or microorganisms such as fungi and bacteria with different biological activities (Ortiz and Sansinenea 2018). DEHP isolated from *Calotropis gigantea* plant exhibits antimicrobial activity against *E. coli, S. aureus*, *Bacillus subtilis*, *Shigella dysenteriae Shigella shiga*, *Shigella sonnei*, *Sarcina lutea,* and *A. flavus* (Habib and Karim 2009). Fungi can produce this compound. DEHP was recently isolated from *Aspergillus awamori* and displayed antifungal and antibacterial activities against *Candida albicans* and Gram-positive bacteria *Sarcina lutea* (Lotfy et al. 2018). *Aspergillus fumigatus* also secrete DEHP (Abdel-Aziz et al. 2018). *Penicillium janthinellum* contains DEHP as a major bioactive compound with antioxidant, antitumor, and antiviral activities (El-Sayed et al. 2015). *Actinomycetes* (filamentous bacteria) can generate this compound. DEHP isolated from *Streptomyces* sp. TN17 showed antimicrobial activities against Gram-positive bacteria and fungi (Smaoui et al. 2011). *Nocardia levis* secretes DEHP that inhibits Gram-positive bacteria and fungi (Kavitha et al. 2009). DEHP is the major component of *n-*hexane extract from fungus combs. Therefore, termites (*M. gilvus* H.) might synthesize DEHP to suppress the growth of another species. *n-*Hexane extract inhibited only Gram-positive bacteria with MIC of 1.56 mg/mL and MBC of 3.13 mg/mL but not fungus *Aspergillus*. Therefore, another compound in fungus comb is responsible for its antifungal activity.

Phenolic compounds are extensively available in plant tissues and have a critical role in highly effective bioactivity. Phenol is a well-known antibacterial agent, i.e., 2-methoxyphenol (guaiacol) and 2,6-dimethoxyphenol (syringol). Guaiacol and syringol isolated from *C. japonica* wood vinegar showed a strong antimicrobial effect against *Pythium splendens, Ralstonia solanacearum*, *Fusarium oxysporum*, and *Phytophthora capsici.* Guaiacol had MIC values of 1.25 mg/mL against *R. solanacearum* and *P. splendens* and 2.5 mg/mL against *P. capsica* and *F. oxysporum* (Hwang et al. 2005)*.* Another study showed the antibacterial activity of *Litchi chinensis* wood vinegar against *S. aureus*, *Acinetobacter baumannii*, and *P. aeruginosa* due to its high phenolic compositions such as 2,6-dimethoxyphenol (29.54%), 2-methoxyphenol (12.36%), and 3,5-dimethoxy-4-hydroxytoluene (11.07%) (Yang et al. 2016). Ethyl acetate extract from fungus combs also consisted of guaiacol, syringol, and DEHP and exhibited antibacterial and antifungal activities against *S. aureus* ATCC 25923, *E. coli* ATCC 25922, *P. aeruginosa* ATCC 2785, *A. flavus* FNCC 6181, and *A. niger* FNCC 6114. Given their presence in ethyl acetate extract from fungus combs at relatively high concentrations, both phenols were regarded as the major antimicrobial constituents in fungus comb. Furthermore, guaiacol and syringol are generally recognized as safe by the Flavor Extract Manufacturers Association. According to Joint FAO/WHO Expert Committee on Food Additives, these compounds are safe for food application. Therefore, ethyl acetate extract from fungus combs shows potential use for food preservation, such as in food packaging. Its application as an antimicrobial agent in packaging material must be further investigated.

## Supplementary Information

**Additional file 1: Figure S1.** MIC of fungus comb extracts against *Escherichia coli* ATCC 25922, *Staphylococcus aureus ATCC* 25923 and *Pseudomonas aeruginosa AT*CC 27853. **Figure S2.** MBC of fungus comb extracts against *Escherichia coli* ATCC 25922, *Staphylococcus aureus ATCC *25923 and *Pseudomonas aeruginosa AT*CC 27853. **Figure S3.** MIC of fungus comb extracts against *Aspergillus flavus* FNCC 6181 and *Aspergillus niger* FNCC 6114. **Figure S4.** MFC of fungus comb extracts against *Aspergillus flavus* FNCC 6181 and *Aspergillus niger* FNCC 6114. **Figure S5.** Chromatogram of GC–MS analysis of the ethyl acetate (**a**) n-hexane (**b**) extracts. **Table S1**. The chemical composition of fungus comb ethyl acetate extract. **Table S2**. The chemical composition of fungus comb n-hexane extract.

## Data Availability

The authors declare that data supporting the findings of this study are available within the article and its supplementary information files.

## Abbreviations

*E. coli*: *Escherichia coli*
*P. aeruginosa*: *Pseudomonas aeruginosa*
*S. aureus*: *Staphylococcus aureus*
*A. flavus*: *Aspergillus flavus*
*A. niger*: *Aspergillus niger*
MIC: Minimum inhibitory concentration
MBC: Minimum bactericidal concentration
MFC: Minimum Fungicidal Concentration
DIZ: Diameter Inhibition Zone
Amp: Ampicillin
Cipro: Ciprofloxacin
DEHP: Bis(2-ethylhexyl) phthalate
Guaiacol: 2-Methoxy-phenol
Syringol: 2:6-Dimethoxyphenol
ND: Not detected
NA: Not available
