# Supplementary material for: Antimicrobial activities of fungus comb extracts isolated from Indomalayan termite (Macrotermes gilvus Hagen) mound
Source: AMB Express. 2022 Feb 10;12:14. doi: 10.1186/s13568-022-01359-0 (PMC8831673; doi:10.1186/s13568-022-01359-0)
Supplement: Supplementary file 1 — Additional file 1: Figure S1. MIC of fungus comb extracts against Escherichia coli ATCC 25922, Staphylococcus aureus ATCC 25923 and Pseudomonas aeruginosa ATCC 27853. Figure S2. MBC of fungus comb extracts against Escherichia coli ATCC 25922, Staphylococcus aureus ATCC 25923 and Pseudomonas aeruginosa ATCC 27853. Figure S3. MIC of fungus comb extracts against Aspergillus flavus FNCC 6181 and Aspergillus niger FNCC 6114. Figure S4. MFC of fungus comb extracts against Aspergillus flavus FNCC 6181 and Aspergillus niger FNCC 6114. Figure S5. Chromatogram of GC–MS analysis of the ethyl acetate (a) n-hexane (b) extracts. Table S1. The chemical composition of fungus comb ethyl acetate extract. Table S2. The chemical composition of fungus comb n-hexane extract. [file 13568_2022_1359_MOESM1_ESM.docx]

**ADDITIONAL FILE 1**

# **Antimicrobial activities of fungus comb extracts isolated from Indomalayan termite**

# **(*Macrotermes gilvus* Hagen) mound**

**Applied Microbiology and Biotechnology Express**

Lucia Dhiantika Witasari^*^, Khairunnasa Wizdjanul Wahyu, Bonifasia Junita Anugrahani, Dina Clarissa Kurniawan, Aris Haryanto, Dodi Nandika, Lina Karlinasari, Arinana, Irmanida Batubara, Djoko Santoso, Yanti Rachmayanti , Dikhi Firmasyah, I Ketut Sudiana, Decsa Medika Hertanto.

*Corresponding author:

Lucia Dhiantika Witasari

Address : Department of Food and Agricultural Product Technology, Faculty of Agricultural Technology, Gadjah Mada University, Bulaksumur, Yogyakarta, 55281, Indonesia

email : [dhiantea_k@ugm.ac.id](mailto:dhiantea_k@ugm.ac.id).

Orchid ID : 0000-0001-9614-4540

Figure S1. MIC of fungus comb extracts against *Escherichia coli* ATCC 25922, *Staphylococcus aureus ATCC 25923* and *Pseudomonas aeruginosa AT*CC 27853

Figure S2. MBC of fungus comb extracts against *Escherichia coli* ATCC 25922, *Staphylococcus aureus ATCC 25923* and *Pseudomonas aeruginosa AT*CC 27853.

Figure S3. MIC of fungus comb extracts against *Aspergillus flavus* FNCC 6181 and *Aspergillus niger* FNCC 6114.

Figure S4. MFC of fungus comb extracts against *Aspergillus flavus* FNCC 6181 and *Aspergillus niger* FNCC 6114.

Figure S5. Chromatogram of GC-MS analysis of the ethyl acetate (a) n-hexane (b) extracts

Table S1. The chemical composition of fungus comb ethyl acetate extract.

Table S2. The chemical composition of fungus comb n-hexane extract


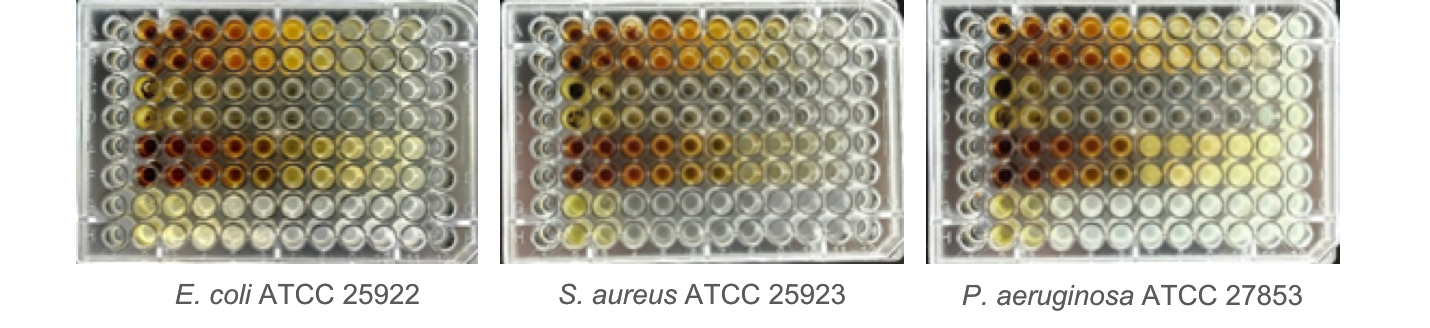


Figure S1. MIC of fungus comb extracts against *Escherichia coli* ATCC 25922, *Staphylococcus aureus ATCC 25923* and *Pseudomonas aeruginosa AT*CC 27853

A,B, ethyl acetate extract; C,D, water extract; E,F, methanol extract; G,H, n-hexane extract; 1, negative control; 2, 100 mg/ml; 3, 50 mg/ml; 4, 25 mg/ml; 5, 12 mg/ml; 6, 6.25 mg/ml; 7, 3.125 mg/ml; 8, 1.56 mg/ml; 9, 0.78 mg/ml; 10, 0.39 mg/ml; 11, 0.19 mg/ml; 12, positive control


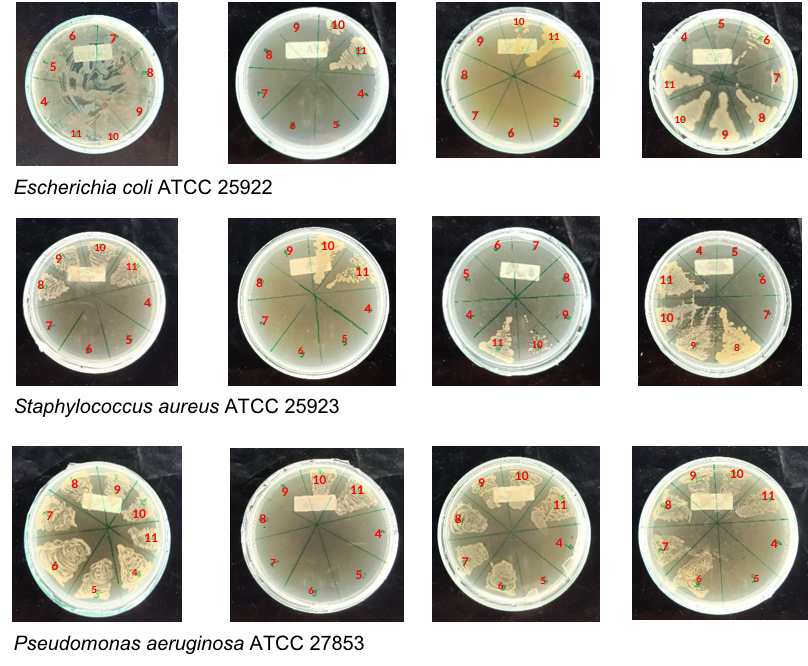


Figure S2. MBC of fungus comb extracts against *Escherichia coli* ATCC 25922, *Staphylococcus aureus ATCC 25923* and *Pseudomonas aeruginosa AT*CC 27853.

a, n-hexane extract; b, ethyl acetate extract; c, methanol extract; d, water extract. 4, 25 mg/ml; 5, 12 mg/ml; 6, 6.25 mg/ml; 7, 3.125 mg/ml; 8, 1.56 mg/ml; 9, 0.78 mg/ml; 10, 0.39 mg/ml; 11, 0.19 mg/ml.


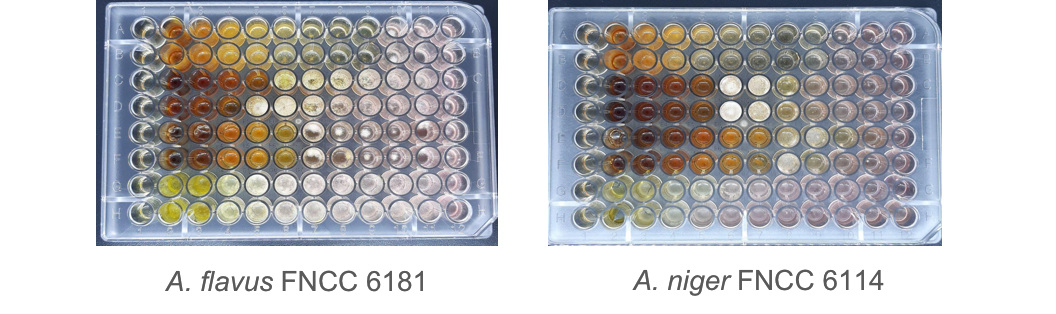


Figure S3. MIC of fungus comb extracts against *Aspergillus flavus* FNCC 6181 and *Aspergillus niger* FNCC 6114.

A,B, ethyl acetate extract; C,D, water extract; E,F, methanol extract; G,H, n-hexane extract; 1, negative control; 2, 100 mg/ml; 3, 50 mg/ml; 4, 25 mg/ml; 5, 12 mg/ml; 6, 6.25 mg/ml; 7, 3.125 mg/ml; 8, 1.56 mg/ml; 9, 0.78 mg/ml; 10, 0.39 mg/ml; 11, 0.19 mg/ml; 12, positive control


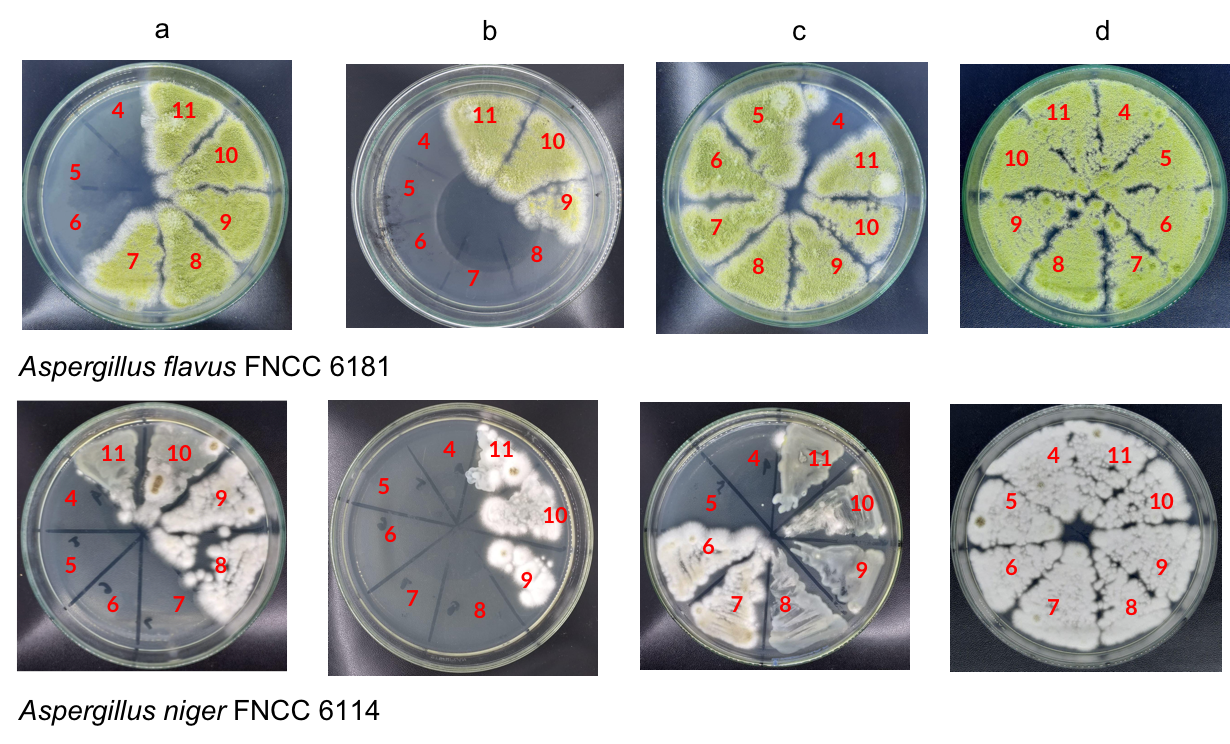


Figure S4. MFC of fungus comb extracts against *Aspergillus flavus* FNCC 6181 and *Aspergillus niger* FNCC 6114.

a, n-hexane extract; b, ethyl acetate extract; c, methanol extract; d, water extract. 4, 25 mg/ml; 5, 12 mg/ml; 6, 6.25 mg/ml; 7, 3.125 mg/ml; 8, 1.56 mg/ml; 9, 0.78 mg/ml; 10, 0.39 mg/ml; 11, 0.19 mg/ml.

a.


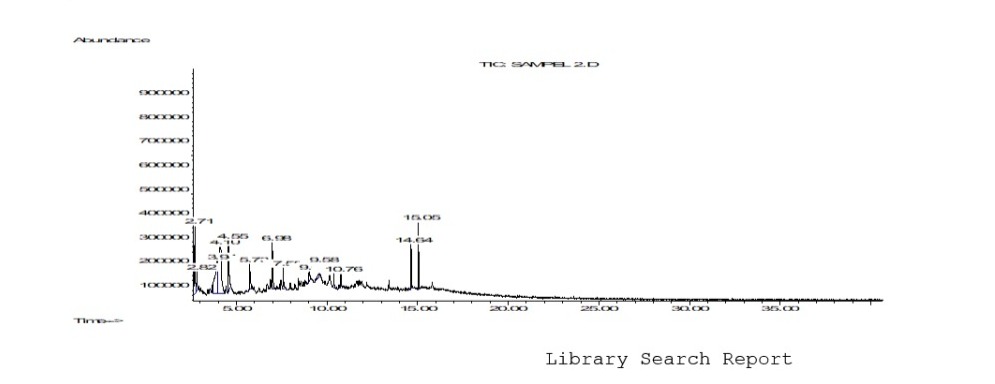


b.


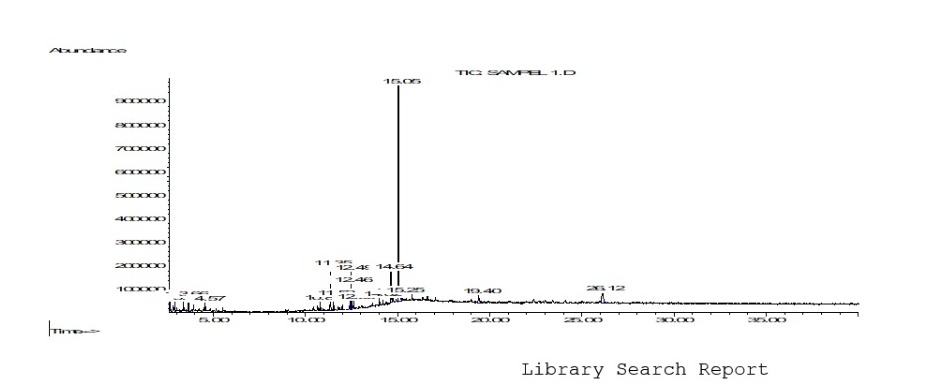


Figure S5. Chromatogram of GC-MS analysis of the ethyl acetate (a) n-hexane (b) extracts

The spectrum data on each peak were compared with the data in the WILEY 9th library.

Table S1. The chemical composition of fungus comb ethyl acetate extract.

| **Compound** | **Relative content (%)** | **Reference** |
| --- | --- | --- |
| Glycerol | 28.93 | Nandika *et al.,* 2021 |
| Phenol, 2-methoxy- | 8.54 |  |
| Phenol, 2,6.dimethoxy- | 6.55 |  |
| Bis(2-ethylhexyl) phthalate | 4.82 |  |

Table S2. The chemical composition of fungus comb n-hexane extract

| **Compound** | **Relative content (%)** | **Reference** |
| --- | --- | --- |
| Bis(2-ethylhexyl) phthalate | 69.43 |  |
| Methyl palmitate | 4.55 | Nandika *et al.,* 2021 |
| Methyl oleate | 4.17 |  |
| Methyl linolelaidate | 2.03 |  |
| Benzenepropanoic acid, 3,5-bis(1,1 dimethylethyl)-4-hydroxy-,methyl ester | 1.16 |  |
|  |  |  |
